# Intravitreal Brolucizumab for Choroidal Neovascularization Associated to Angioid Streaks

**DOI:** 10.1155/2022/3442306

**Published:** 2022-07-14

**Authors:** Somnath Chakraborty, Jay Umed Sheth

**Affiliations:** ^1^Retina Institute of Bengal, Siliguri, India; ^2^Surya Eye Institute and Research Center, Mumbai, India

## Abstract

A 44-year-old-female with angioid streak- (AS-) associated choroidal-neovascularization (CNV) was treated with one dose of intravitreal brolucizumab (IB). At one-month, the patient's visual acuity (VA) improved from 20/120 to 20/40 with a dry macula on spectral-domain optical-coherence tomography (SD-OCT). After observation, the VA improved further to 20/32 with absence of any fluid on the SD-OCT at three months. No ocular or systemic adverse events were noted. In conclusion, intravitreal brolucizumab (IB) is an efficacious and safe therapeutic option for the management of CNV secondary to AS. Further prospective studies with a larger sample size, varied therapeutic regimens, and longer follow-up period are needed to corroborate our findings.

## 1. Introduction

Angioid streaks (AS) are linear cracks or dehiscence in an unusually weak or calcified Bruch's membrane [1]. They can be caused by systemic diseases such as pseudoxanthoma elasticum (PXE), Ehlers–Danlos syndrome, Paget's disease, or sickle cell disease, although up to 50% of patients may have no obvious systemic association [1]. One of the most significant complications of AS is choroidal neovascularization (CNV), which has been recorded in 70–86% of eyes [1, 2]. Over time, 71% of these patients also develop CNV in the contralateral eye [1, 2]. Subfoveal and juxtafoveal CNV in AS have been treated with photodynamic therapy (PDT) with suboptimal visual outcomes in the long term [3, 4]. Similarly, equivocal results with considerable recurrences have been observed in extrafoveal CNV treated with thermal laser (TL) photocoagulation [5, 6].

According to recent research, the use of antivascular endothelial growth factor (anti-VEGF) therapy is efficacious in the management of CNV secondary to AS [7–12]. The use of ranibizumab, bevacizumab, and aflibercept, among the existing anti-VEGF medications, has been documented for treating AS-related CNV [7–12]. The most recently FDA-approved treatment for neovascular age-related macular degeneration (nAMD) is the brolucizumab (Beovu ®; Novartis, Basel, Switzerland) molecule [13]. It has been successfully used in the management of recalcitrant diabetic macular edema (DME) as an off-label medication [13].

We herein report a novel case of AS-associated CNV which was successfully treated with intravitreal brolucizumab (IB).

## 2. Case Report

**Figure 1:**
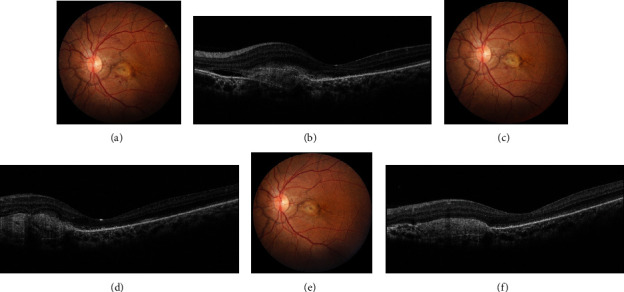
At baseline, the color fundus photograph (CFP) of the left eye (OS) showed the presence of angioid streaks (AS) with subretinal hemorrhage (SRH) suggestive of an underlying choroidal neovascularization (CNV) (a). Spectral-domain optical coherence tomography of the OS illustrated the presence of subretinal fluid (SRF) with subretinal hyperreflectivity and thickened Bruch's membrane (b). After undergoing intravitreal brolucizumab injection, the SRH reduced significantly at one month (c) and disappeared completely by three months (e) as noted on the CFP. The SD-OCT scans at months 1 (d) and 3 (f) demonstrated complete resolution of the fluid and a reduction in the subretinal hyperreflectivity.

A 44-year-old female presented with a reduction in vision and metamorphopsia in the left eye (OS) for 3 months. Her best-corrected visual acuity (BCVA) was 20/20 in the right eye (OD) and 20/120 in OS. Both eyes (OU) anterior segments were unremarkable with normal intraocular pressure (IOP) of 18 mmHg. Fundus examination of OD was normal while OS showed the presence of AS with subretinal hemorrhage (SRH). On spectral-domain optical coherence tomography (SD-OCT), there was the presence of subretinal hyperreflectivity, subretinal fluid (SRF), and a thickened Bruch's membrane. A diagnosis of CNV secondary to AS was made and the patient underwent treatment with intravitreal brolucizumab (IB) (6 mg/0.5 ml) following a discussion of all the therapeutic options. At one month, the patient's BCVA improved to 20/40 with a dry macula on SD-OCT. Trace SRH was present clinically, and the patient was observed. The trace SRH resolved completely by the end of three months with an improvement in BCVA to 20/32. The SD-OCT was dry at this stage too. There were no ocular or systemic adverse effects observed.  Figure 1 depicts the changes in color fundus photograph (CFP) and SD-OCT in OU throughout the course of the treatment.

## 3. Discussion

In our case report, we describe a patient with AS who developed CNV which was successfully treated by brolucizumab injection. To date, although off-label, the use of brolucizumab for CNV management in AS has not been reported. In our case, we found no ocular or systemic adverse effects.

The AS is histologically characterized as cracks in the elastic lamina of the Bruch's membrane (BM) with accompanying calcification [1, 14]. The majority of AS are asymptomatic and need periodic fundus examination [1, 2]. AS can cause substantial vision impairment due to CNV development [2]. The risk factors for the development of CNV include advancing age and the length, width, and location of the AS [2, 14, 15]. Wider and longer AS have been linked to an increased likelihood of CNV in several studies [2, 15]. Moreover, if the AS is situated within one optic disc diameter of the foveola, the probability of CNV formation is significantly increased [15, 16]. Various therapeutic modalities, including PDT and TL, have been explored in the past but are no longer employed due to poor outcomes [3–6, 17]. After VEGF inhibitors were established in the treatment of CNV secondary to nAMD, they provided an effective therapeutic option for other chorioretinal disorders such as AS [1, 2].

Cases with AS and associated CNV are infrequent, and hence, there have been very few studies evaluating the efficacy of intravitreal anti-VEGF therapy in its management. Teixeira et al. initially reported the use of intravitreal bevacizumab injection in a patient with subfoveal CNV and AS [18]. Subsequently, the use of the other anti-VEGF molecules, including ranibizumab and aflibercept, has been reported in the literature [7, 8, 11, 12]. A retrospective series of 35 eyes with CNV secondary to AS treated with ranibizumab was evaluated by Mimoun et al. [19]. At a mean follow-up of 24.1 months, the authors noted an improvement or stabilization of BCVA in 30 eyes (85.7%), absence of leakage on fundus fluorescein angiography (FFA) in 23 eyes (65.7%), and reduction or stabilization of central macular thickness (CMT) in 18 eyes (51.5%) [19]. The Italian EYLEA-STRIE study was a multicentre, open-label, phase IIb study evaluating the safety and efficacy of intravitreal aflibercept therapy on a pro-re-nata basis in 23 eyes of 20 patients with CNV secondary to AS [11]. At 48 weeks, the authors observed an improvement in BCVA with visual stabilization within 15 letters in 81.8% of the eyes and a significant reduction in CMT [11]. According to Sekfali et al., switching to aflibercept injection was a good treatment choice for patients with CNV secondary to AS who had resistant or recurrent illness after at least 12 months of ranibizumab therapy [20]. The authors demonstrated an improvement in BCVA with a significant reduction in CMT, along with 71% of the eyes having no intraretinal (IRF)/SRF and 77% having no leakage on FFA among the 14 eyes [20]. However, the efficacy of brolucizumab injection in these cases, whether treatment-naïve or with recalcitrant/recurrent disease, has not been reported in the literature.

Brolucizumab is a single-chain humanized antibody fragment with a relatively small molecular size and a weight of only 26 kDa [13]. Brolucizumab's effective molar dose is 12-fold greater than aflibercept and 22-fold more than ranibizumab due to its low molecular weight, which is 1/1.8 of that of ranibizumab and 1/4 of that of aflibercept [13]. Smaller molecules, such as brolucizumab, have exhibited superior target-tissue penetration, resulting in longer durability, better efficacy, and reduced systemic exposure [21]. These molecular properties of brolucizumab can be advantageous in a condition such as AS which has thickened and calcified Bruch's membrane and an abnormal retinal pigment epithelium (RPE) [1, 14]. Theoretically, its smaller size and increased tissue penetration make it an ideal candidate to pass through this abnormal RPE-Bruch's membrane tissue complex and act on the CNV. With this backdrop, our patient received intravitreal brolucizumab (IB) and demonstrated promising visual and anatomical outcomes. Moreover, although intraocular inflammation (IOI) remains a concern with brolucizumab therapy, we did not note any ocular or systemic adverse event in our case up to three months [13].

In conclusion, this is the first reported case demonstrating the role of intravitreal brolucizumab injection in the management of CNV secondary to AS. Further prospective studies with a larger sample size and different therapeutic regimens are needed to validate our findings.
